# Aptamer Selection Based on G4-Forming Promoter Region

**DOI:** 10.1371/journal.pone.0065497

**Published:** 2013-06-04

**Authors:** Wataru Yoshida, Taiki Saito, Tomomi Yokoyama, Stefano Ferri, Kazunori Ikebukuro

**Affiliations:** 1 Department of Biotechnology and Life Science, Tokyo University of Agriculture & Technology, Koganei, Tokyo, Japan; 2 Japan Science and Technology Agency, CREST, Koganei, Tokyo, Japan; University of Helsinki, Finland

## Abstract

We developed a method for aptamer identification without *in vitro* selection. We have previously obtained several aptamers, which may fold into the G-quadruplex (G4) structure, against target proteins; therefore, we hypothesized that the G4 structure would be an excellent scaffold for aptamers to recognize the target protein. Moreover, the G4-forming sequence contained in the promoter region of insulin can reportedly bind to insulin. We thus expected that G4 DNAs, which are contained in promoter regions, could act as DNA aptamers against their gene products. We designated this aptamer identification method as “G4 promoter-derived aptamer selection (G4PAS).” Using G4PAS, we identified vascular endothelial growth factor (VEGF)165, platelet-derived growth factor-AA (PDGF)-AA, and RB1 DNA aptamers. Surface plasmon resonance (SPR) analysis revealed that the dissociation constant (*K*_d_) values of VEGF165, PDGF-AA, and RB1 DNA aptamers were 1.7 × 10^−7^ M, 6.3 × 10^−9^ M, and 4.4 × 10^−7^ M, respectively. G4PAS is a simple and rapid method of aptamer identification because it involves only binding analysis of G4 DNAs to the target protein. In the human genome, over 40% of promoters contain one or more potential G4 DNAs. G4PAS could therefore be applied to identify aptamers against target proteins that contain G4 DNAs on their promoters.

## Introduction

Aptamers are nucleic acid ligands that specifically bind to target molecules [1], [2]. Aptamers have several advantages over antibodies as molecular recognition elements; therefore, many aptamer-based sensors have been reported [3], [4]. Moreover, the vascular endothelial growth factor (VEGF) RNA aptamer has been approved for the treatment of neovascular age-related macular degeneration [5], indicating that aptamer identification is important for applications of aptamers. In general, aptamers are selected from a random nucleic acid library *in vitro*, designated as Systematic Evolution of Ligands by EXponential enrichment (SELEX). In SELEX, oligonucleotides bound to the target are eluted and then polymerase chain reaction (PCR)-amplified to prepare the next round library. After several rounds of selection, the enriched library is sequenced and aptamers are then identified. Although SELEX is an efficient method for screening aptamers, it sometimes fails to obtain aptamers because of PCR bias [6], [7] and the limited diversity of the library used in experimental manipulation [8]. Therefore, the development of a method for aptamer selection without SELEX is required.

Identification of riboswitches is one the method for obtaining RNA aptamers without SELEX [9], [10]. Riboswitches are RNA elements that sense metabolites to control the corresponding metabolic gene expression. Riboswitches comprise a sensing domain and regulating domain. The sensing domains are regarded as RNA aptamers against the corresponding metabolites. However, this strategy is only applied to identify RNA aptamers against small-molecule metabolites. Chushak has also proposed a virtual screening method for aptamer identification *in silico* [11]. The virtual screening method would be powerful; however, it also requires further *in vitro* selection to identify aptamers. Moreover, the method is limited to aptamer selection for small molecules.

Aptamers fold into several secondary structures such as the stem–loop, pseudoknot, three-way junction, and G-quadruplex (G4) structures. Among these structures, the G4 structure is the most adopted by many aptamers [12]. G4 structures are four-stranded DNA structures that consist of planar arrays of four guanines and intervening loops. The length of the intervening loops and number of planar arrays are variable, indicating that the G4 structure has large diversity. We have suggested that G4 tends to preferentially bind to the β-structures of proteins [13]. Moreover, G4 is considered to be an excellent motif to interact with the cationic domain of protein because G4 has twice the negative charge density of double helices [14]. Therefore, we hypothesized that G4 DNA would be an excellent motif for aptamers to recognize target proteins. In particular, we obtained DNA aptamers that were expected to fold into the G4 structure against thrombin [15], DNA polymerase [16], insulin [17], pyrroquinoline quinone glucose dehydrogenase (PQQGDH) [18], VEGFA [19], flavin adenine dinucleotide-dependent glucose dehydrogenase (FADGDH) [20], and α-synuclein oligomers [13].

In the genome, G4 sequences have been identified in telomeres and some promoter regions, e.g., VEGFA [21], platelet-derived growth factor A (PDGFA) [22], retinoblastoma 1 (RB1) [23], c-KIT [24], [25], c-MYC [26], Insulin [27]–[29], KRAS [30], B-cell lymphoma 2 (BCL-2) [31], hypoxia-inducible factor 1α (HIFIα) [32], transcription factor MYB [33], PDGF receptor β (PDGFRβ) [34], and human telomerase reverse transcriptase (TERT) [35]. Moreover, *in silico* analysis demonstrated that over 40% promoters contain one or more potential G4-forming sequences [36]–[39]. In several promoters, G4-binding proteins have been identified, and the mechanism of gene regulation by G4 has been proposed. This suggested that the G4 structure is an important element for transcriptional regulation. Moreover, the G4-forming sequence in the insulin promoter (ILPR2) has been shown to have the ability to bind to both insulin and IGF2 [27]–[29]. The insulin gene promoter region contains three variants of ILPR2, and the G4 topology of the variants reflects their ability to bind to the proteins. These results suggested that insulin and IGF2 are recognized by particular G4-forming DNAs. We thus assumed that G4 DNAs, which are contained in promoter regions, would serve as DNA aptamers against proteins whose expression is regulated by G4 DNA. This suggests that DNA aptamers may be obtained from genomic sequence without SELEX. We designated this aptamer identification method “G4 promoter-derived aptamer selection (G4PAS).”

To demonstrate that DNA aptamers can be obtained by G4PAS, we analyzed the binding ability of G4 DNA from the promoter regions of VEGFA, PDGFA, RB1, and c-KIT against VEGFA (VEGF165), PDGF-AA, RB1, and c-KIT, respectively. We also analyzed the binding specificity of these G4 DNAs.

## Materials and Methods

### Materials

Recombinant human VEGFA (VEGF165 and VEGF121) and recombinant human PDGF-AA homodimers were purchased from R&D Systems (Minneapolis, USA). Recombinant human RB1 and the intracellular domain of recombinant human c-KIT (corresponding to amino acids 544–976) were purchased from Abcam (Cambridge, UK). The extracellular domain of recombinant human c-KIT (corresponding to amino acids 1–516) was purchased from Sino Biological (Beijing, China). FITC-labeled VEGF G4, c-KIT G4-1, and c-KIT G4-2 were purchased from Life Technologies (CA, USA) and TAMRA-labeled PDGFA G4 and RB1 G4 were purchased from Greiner Bio-one (Frickenhausen, Germany).

### Circular Dichroism Spectroscopy

All DNA samples were diluted to 2 µM in TK buffer (10 mM Tris–HCl, 100 mM KCl, pH 7.5). These DNA samples were denatured at 95°C for 10 min and then allowed to cool to room temperature for 30 min. Circular dichroism (CD) spectra were measured using a J-820 spectropolarimeter (JASCO, Tokyo, Japan) and a quartz cell of 10 mm optical path length (Agilent, CA, USA) at 25°C.

### Gel Shift Assay

The gel shift binding assay was performed using 5′-FITC- or 5′-TAMRA-modified oligonucleotides. Prior to use, all the oligonucleotides were denatured in a binding buffer (10 mM Tris–HCl, 150 mM NaCl, 5 mM KCl; pH 7.4) at 95°C for 10 min and then allowed to cool to room temperature for 30 min. The oligonucleotides were incubated with target proteins for 30 min at room temperature. Concentrations of oligonucleotides and target proteins used were as follows: 0.5 µM of VEGFA G4; 0.5 µM of PDGFA G4; 1 µM of RB1 G4; 1 µM of c-KIT G4-1 and G4-2; 2.6 µM of VEGF165; 2.6 µM of VEGF121; 1.8 µM of PDGF-AA; 0.5 µM of RB1; and 1 µM of the intracellular and extracellular domains of c-KIT. Five microliters of the mixtures were electrophoresed on 12% polyacrylamide gel in Tris/borate/EDTA (TBE buffer), followed by scanning the gel using Typhoon8600 (GE Healthcare).

The gel shift competition assay was performed using 5′-FITC-labeled VEGFA G4 and non-labeled PDGFA G4 and RB1 G4. All the oligonucleotides were separately folded by heat treatment as described above. The oligonucleotides (0.5 µM) and VEGFA (2.6 µM) were mixed, and then native polymerase gel electrophoresis (PAGE) was performed as described above.

### Surface Plasmon Resonance (SPR) Assay

For binding analysis of the oligonucleotides to VEGF165 or PDGF-AA, approximately 7500 RU of VEGF165 or 1900 RU of PDGF-AA was immobilized on a sensor chip CM5 (GE Healthcare) using an amine coupling procedure in VEGF165 immobilization buffer (10 mM acetate; pH 6.0) or PDGF-AA immobilization buffer (10 mM HEPES, 150 mM NaCl, 5 mM KCl; pH 7.0), respectively. The non-labeled oligonucleotides were folded by heat treatment in a binding buffer (10 mM Tris–HCl, 150 mM NaCl, 5 mM KCl; pH 7.4) as described above. Various concentrations of oligonucleotides were injected onto the VEGF165- or PDGF-AA-immobilized chip at a flow rate of 30 µl/min at 25°C. The VEGF165-immobilized chip was regenerated by injection of a mixture of 1 M NaCl and 1 mM NaOH. The PDGF-AA-immobilized chip was regenerated by injection of 1 M NaCl. The dissociation constant (*K*_d_) values were calculated by fitting the association and dissociation rates using BIA evaluation software (GE Healthcare).

The ability of oligonucleotides to bind to RB1 was evaluated by measuring SPR on the sensor chip SA in a Biacore T200 (GE Healthcare). The 5′-biotinylated oligonucleotides were folded by heat treatment in RB1 binding buffer (10 mM Tris–HCl, 650 mM NaCl, 5 mM KCl; pH 7.4) as described above. The sensor chip SA was washed three times with a mixture of 1 M NaCl and 50 mM NaOH, and approximately 500 RU of oligonucleotides were immobilized on it. Various concentrations of RB1 protein were injected onto the oligonucleotide-immobilized chip at a flow rate of 30 µl/min at 25°C. The *K*_d_ values were calculated by Scatchard plot analysis.

## Results

### Binding Analysis of G4-forming DNA in Promoter Regions to Target Proteins

**Figure 1:**
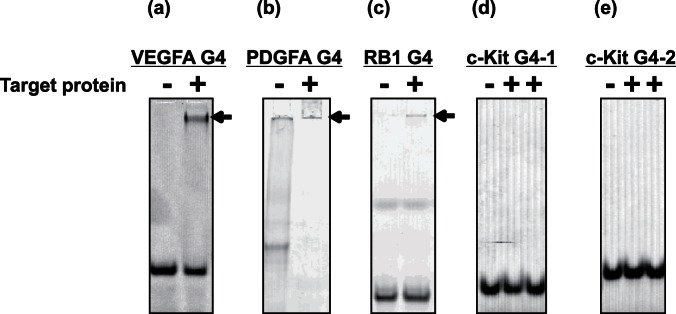
Gel shift binding assay of G4-forming DNAs to target proteins. In the presence or absence of targets, fluorescent-labeled G4-forming DNAs were electrophoresed on 12% polyacrylamide gel in TBE buffer, and fluorescence images were then detected. Arrows indicate bands of DNA–protein complex. Gel shift assay of VEGFA G4 to VEGF165 (a), PDGFA G4 to PDGF-AA (b), RB1 G4 to RB1 (c), c-KIT G4-1 to c-KIT (d), and c-KIT G4-2 to c-KIT (e). In the assay of c-KIT G4-1 and c-KIT G4-2, both the intracellular (second lane) and extracellular (third lane) domains of c-KIT were used.

Several promoter regions reportedly contain G4-forming DNA. To investigate whether the G4-forming DNAs bind to their protein products *in vitro*, we focused on the VEGFA, PDGFA, RB1, and c-KIT genes. The VEGFA promoter contains one parallel G4 structure located −50 to −85 bp upstream of the transcription start site (TSS), the PDGFA promoter contains one parallel G4 structure located −47 to −82 bp upstream of TSS, the RB1 gene contains one antiparallel G4 structure located +169 to +186 bp downstream of TSS, and the c-KIT promoter contains two G4 structures located −74 to −94 bp, and −23 to −44 bp upstream of TSS. We designated these G4 DNAs as VEGFA G4, PDGFA G4, RB1 G4, and c-KIT G4-1 and c-KIT G4-2, respectively (Table 1). To perform the gel shift assay, these fluorescent-labeled G4 DNAs were synthesized. We confirmed that these fluorescence-labeled DNAs form a parallel G-quadruplex structure, which is similar to that formed by non-labeled DNAs, as determined by measurement of CD spectra (Figure S1). We used the primary isoform of VEGFA (VEGF165) and PDGF-AA as the target proteins of VEGFA G4 and PDGFA G4, respectively. We also used the intracellular and extracellular domains of c-KIT proteins as the target proteins of c-KIT G4 because c-KIT is a membrane protein. In the gel shift assay, we observed a band shift of VEGFA G4, PDGFA G4, and RB1 G4 but not of c-KIT G4s (Figure 1). At the position of the shifted band of these oligonucleotides, we also detected these proteins by silver staining. These results indicated that VEGFA G4, PDGFA G4, and RB1 G4 bound to VEGF165, PDGF-AA, and RB1 protein *in vitro*, respectively; however, c-KIT G4-1 and G4-2 DNAs did not bind to the intracellular and extracellular domains of c-KIT protein. We observed both monomeric and multimeric PDGFA G4 in the absence of PDGF-AA; however, the band of monomeric PDGFA G4 was completely shifted in the presence of PDGF-AA, suggesting that monomeric PDGFA G4 would bind to PDGF-AA**.**

**Table 1:** Oligonucleotides used in this study.

Name	Sequence (5′ to 3′)
VEGFA G4	GGGGCGGGCCGGGGGCGGGGTCCCGGCGGGGCGG
VEGFA G4 Mut.	GTTTCTTTCCGGTTTCGTTTTCCCGGCGGGGCGG
PDGFA G4	GGAGGCGGGGGGGGGGGGGCGGGGGCGGGGGCGGGGGAGGGGCGCGGC
PDGFA G4 Mut.	GGAGGCTTTTTTTTTTTTTCTTTTTCTTTTTCTTTTTATTTTCGCGGC
RB1 G4	CGGGGGGTTTTGGGCGGC
RB-1G4 Mut.	CGTTTTGTTTTGGGCGGC
c-KIT G4-1	CGGGCGGGCGCGAGGGAGGGG
c-KIT G4-2	AGGGAGGGCGCTGGGAGGAGGG
TBA	GGTTGGTGTGGTTGG

### Binding Kinetics of VEGFA G4, PDGFA G4, and RB1 G4 to Target Proteins

**Figure 2:**
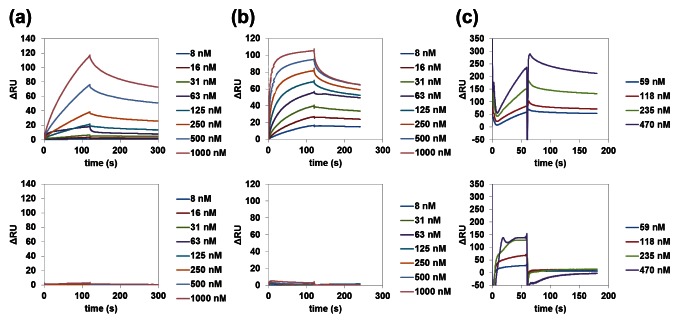
SPR analysis of binding of G4-forming DNAs to target proteins. (a) SPR binding signal of VEGFA G4 (top) and VEGFA G4 mutant (bottom) to VEGF165 immobilized on a CM5 chip. (b) SPR binding signal of PDGFA G4 (top) or PDGFA G4 mutant (bottom) to PDGF-AA immobilized on a CM5 chip. (c) SPR binding signal of RB1 to RB1 G4 immobilized on an SA chip (top) or RB1 G4 mutant immobilized on an SA chip (bottom).

We next investigated the binding kinetics of VEGFA G4, PDGFA G4, and RB1 G4 to their target proteins by SPR. In SPR analysis of VEGFA G4 and PDGFA G4, the target proteins were immobilized on a CM5 chip via amine coupling and non-labeled oligonucleotides were then applied to the sensor chip. In SPR analysis of RB1 G4, biotinylated oligonucleotides were immobilized on the sensor chip SA and RB1 protein was then applied to the sensor chip. As a control, we used mutant VEGFA G4, PDGFA G4, and RB1 G4 that were not expected to form the G4 structure (Table 1). In SPR analysis, we detected the binding signal of VEGFA G4, PDGFA G4, and RB1 G4 to the target proteins, and their *K*_d_ values were calculated to be 1.7×10^−7^ M, 6.3×10^−9^ M, and 4.4×10^−7^ M, respectively (Figure 2). The *K*_d_ value of VEGFA G4 to VEGF165 was similar to that of the VEGF165 DNA aptamer obtained by SELEX [40], and PDGFA G4 bound to PDGF-AA at a nanomolar level, suggesting that DNA aptamers that bind to the target protein with high affinity can be obtained by G4PAS. However, mutant VEGFA G4, PDGFA G4, and RB1 G4 did not bind to their target proteins. These results indicated that the G4 structures are important for recognizing their target proteins.

### Binding Specificity of VEGFA G4, PDGFA G4, and RB1 G4

To analyze the binding specificity of these G4 DNAs, we selected VEGF165 and PDGF-AA as the target proteins because these proteins belong to the superfamily of VEGF/PDGF proteins [41]. To analyze the binding specificity, we conducted SPR measurements. We immobilized VEGF165 and PDGF-AA on CM5 chips via amine coupling and then applied VEGFA G4, PDGFA G4, and RB1 G4 to the sensor chip. We also used a 15-mer thrombin-binding aptamer (TBA), which folds into the G4 structure, as a control [42]. On the VEGF165-immobilized chip, we detected the binding signal of PDGFA G4 and RB1 G4 to VEGF165 as well as VEGFA G4 but did not detect the binding signal of TBA to VEGF165 (Table 2, Figure S2). The *K*_d_ values of PDGFA G4 and RB1 G4 to VEGF165 were calculated to be 1.0 × 10^−8^ M and 3.0 × 10^−7^ M, respectively. However, we did not detect the binding signal of VEGFA G4, RB1 G4, and TBA to PDGF-AA (Table 2, Figure S3). These results indicate that PDGF-AA was specifically recognized by PDGFA G4. In contrast, VEGF165 was recognized by VEGFA G4, PDGFA G4, and RB1 G4 but not by TBA.

**Table 2:** Binding specificity of VEGFA G4, PDGFA G4, and RB1 G4 to VEGF165 and PDGF-AA.

	VEGF165			PDGF-AA		
	*K*_d_ (M)	*k*_on_ (1/Ms)	*k*_off_ (1/s)	*K*_d_ (M)	*k*_on_ (1/Ms)	*k*_off_ (1/s)
VEGFA G4	(1.7±1.5)×10^−7^	(6.8±4.5)×10^3^	(8.0±1.0)×10^−4^	N.B.	N.B.	N.B.
PDGFA G4	(1.0±0.6)×10^−8^	(1.4±1.0)×10^5^	(1.7±1.9)×10^−3^	(6.3±3.7)×10^−9^	(3.0±1.0)×10^5^	(1.7±0.7)×10^−3^
RB1 G4	(3.0±1.1)×10^−7^	(1.8±1.3)×10^5^	(4.5±2.6)×10^−2^	N.B.	N.B.	N.B.
TBA	N.B.	N.B.	N.B.	N.B.	N.B.	N.B.

N.B.: Not bound.

Homology analysis of VEGFA G4, PDGFA G4, and RB1 G4 revealed that the G4-forming region of VEGFA G4 was highly conserved in PDGFA G4 (Figure S4) but not in RB1 G4. RB1 G4 recognized VEGF165; however, the dissociation rate constant (*k*_off_) value was remarkably lower than those of VEGFA G4 and PDGFA G4. These results suggest that RB1 G4 may recognize a different site of VEGF165, which is recognized by VEGFA G4 and PDGFA G4.

### VEGFA G4, PDGFA G4, and RB1 G4 Binding Site Analysis Against VEGF165

**Figure 3:**
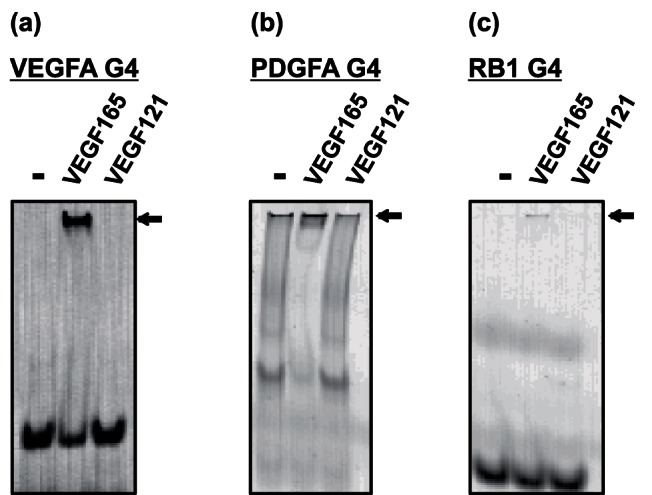
Gel shift binding assay of VEGFA G4, PDGFA G4, and RB1 G4 to VEGF165 and VEGF121. In the presence or absence of the proteins, fluorescent-labeled VEGFA G4, PDGFA G4, and RB1 G4 were electrophoresed on 12% polyacrylamide gel in TBE buffer, and fluorescence images were then detected. Arrows indicate bands of DNA–protein complex. Gel shift assay of VEGFA G4 (a), PDGFA G4 (b), and RB1 G4 (c) to VEGF165 and VEGF121.

VEGFA has several isoforms that are formed by alternative exon splicing. VEGF165 has a receptor-binding domain and a heparin-binding domain. VEGF121, one of the VEGF isoforms, has a common receptor-binding domain to VEGF165 but does not have the heparin-binding domain. The heparin-binding motif contains several basic amino acid residues that are important for interaction with the negatively charged sulfo groups on heparin. We assumed that VEGFA G4, PDGFA G4, and RB1 G4 interact with the heparin-binding domain of VEGF165 via electrostatic interaction. In particular, several aptamers have been identified against heparin-binding proteins such as thrombin [14], [42] and VEGF165 [40]. To investigate whether VEGFA G4, PDGFA G4, and RB1 G4 recognize the heparin-binding domain of VEGF165, we performed a gel shift assay against VEGF165 and VEGF121. In the presence of VEGF165, we confirmed a complex of these G4s with VEGF165 (Figure 3). In contrast, we did not observe any binding activity of these G4s to VEGF121. These results indicate that VEGFA G4, PDGFA G4, and RB1 G4 recognize the heparin-binding domain of VEGF165.

**Figure 4:**
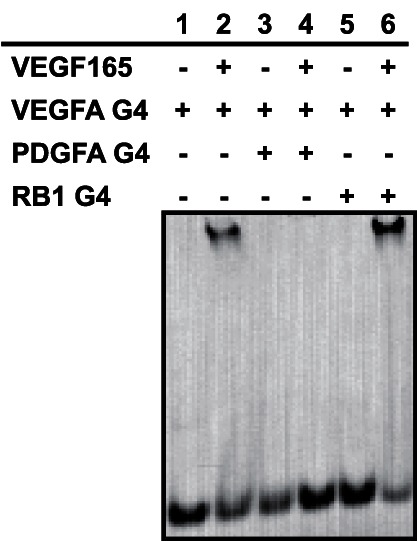
Gel shift competition assay. In the presence of FITC-labeled VEGFA G4 (0.5 µM), non-labeled PGDFA G4 (0.5 µM) or RB1 G4 (0.5 µM) was incubated with VEGF165 (2.6 µM). The mixtures were electrophoresed on 12% polyacrylamide gel in TBE buffer, and FITC images were then detected.

We also performed a gel shift competition assay to investigate whether VEGFA G4, PDGFA G4, and RB1 G4 bind to the same site of VEGF165. In the presence or absence of VEGF165, equal moles of 5′-FITC-labeled VEGFA G4 and non-labeled PDGFA G4 or RB1 G4 were mixed and then analyzed by native PAGE. We did not detect a band shift of VEGFA G4 in the presence of PDGFA G4 (Figure 4). These results suggest that VEGFA G4 and PDGFA G4 recognize the same site on the heparin-binding domain of VEGF165. However, we detected a band shift of VEGFA G4 in the presence of RB1 G4. This result suggests that RB1 G4 would recognize a different site on the heparin-binding domain or that competitive binding was not observed because of the lower binding affinity of RB1 G4 to VEGF165.

## Discussion

G4PAS enabled us to obtain VEGF165, PDGF-AA, and RB1 DNA aptamers with *K*_d_ values of 1.7×10^−7^ M, 6.3×10^−9^ M, and 4.4×10^−7^ M, respectively; however, we did not obtain a c-KIT DNA aptamer. These results demonstrate that although G4PAS cannot be applicable to all proteins, DNA aptamers can be from the G4-forming promoter regions of the target proteins without SELEX. In the human genome, over 40% of promoters contain one or more potential G4-forming sequences, suggesting that these G4 DNAs are potential DNA aptamers against their gene products. As we described, G4PAS cannot be applicable to all proteins; therefore, we need to predict the target proteins to which our strategy can be applied. In this study, we obtained a DNA aptamer against VEGF165 and PDGF-AA that has the heparin-binding domain; the VEGFA G4 aptamer recognized the heparin-binding domain of VEGF165. These results suggest that G4PAS can be applied to proteins with the heparin-binding domain. Heparin-binding domains have been identified in proteases, esterases, growth factors, chemokines, lipid-binding proteins, pathogen proteins, and adhesion proteins [43]; thus, various aptamers may be obtained against these heparin-binding proteins. However, we also obtained an aptamer against RB1 that does not have the heparin-binding domain. Insulin and IGF2 also do not have the heparin-binding domain; thus, further analysis is required to establish the target prediction for G4PAS.

In this report, we analyzed promoter regions that have been reported to form a G4-structure; however, G4PAS could also be applied to other promoter regions. To identify aptamers by G4PAS, we first need to identify putative G4-forming DNAs in target promoter regions. We can obtain a list of human promoter regions containing putative G4-forming DNAs [39] and predict putative G4-forming DNAs in any DNA sequence [44]. Therefore, putative G4-forming DNAs in any target promoter region could be identified as DNA aptamer candidates for G4PAS.

In this study, we obtained aptamers against VEGF165, PDGF-AA, and RB1; however, PDGFA G4 and RB1 G4 also bound to VEGF165. We have reported a sequence mutation method to improve the function of aptamers based on genetic algorithms, designated as “*in silico* maturation (ISM)” [14], [15], [45]–[47]. Using ISM, we improved the binding ability or inhibitory activity of aptamers. We also improved the specificity of aptamers using ISM (unpublished data). Because ISM is not required for *in vitro* selection, we believe that highly specific aptamers may be identified using a combination of G4PAS and ISM without SELEX.

In gene promoters that have the ability to bind to their gene products, gene expression can be controlled by feedback regulation. RB1 G4 and VEGFA G4 can be involved in feedback regulation of the RB1 gene and VEGFA gene, respectively, because RB1 is expressed in the nucleus and VEGFA is translocated to the nucleus in the cells situated at the edges of a wound [48], [49]. On the other hand, PDGFA G4 might not be involved in feedback regulation in human cells, because PDGF-AA is not expressed in the nucleus. Thus, we assumed that the feedback regulation may possibly be used in an ancient state in evolution. Therefore, we believe that the identification of G4 DNAs, which bind to their gene products, could contribute not only aptamer identification but also provide new insights into gene regulation by G4 DNAs.

## Supporting Information

Figure S1 **CD spectra of fluorescence-labeled and non-labeled DNAs.** VEGFA G4 (a), PDGFA G4 (b), RB1 G4 (c), c-KIT G4-1 (d), and c-KIT G4-2 (e) were analyzed.
(EPS)

Figure S2 **SPR analysis of binding of G4 DNAs to VEGF165 immobilized on a CM5 chip.** (a) SPR binding signal of PDGFA G4 to VEGF165; (b) SPR binding signal of RB1 G4 to VEGF165; and (c) SPR binding signal of TBA to VEGF165.
(EPS)

Figure S3 **SPR analysis of binding of G4 DNAs to PDGF-AA immobilized on a CM5 chip.** (a) SPR signal of VEGFA G4 to PDGF-AA; (b) SPR signal of RB1 G4 to PDGF-AA; and (c) SPR signal of TBA to PDGF-AA.
(EPS)

Figure S4 **Homology analysis of VEGFA G4 and PDGFA G4.** Sequences conserved between PDGFA G4 and VEGFA G4 are boxed. The G4-forming region of VEGFA G4 is indicated by asterisks.
(EPS)
